# The Challenge of Unlocking the Biological Secrets of Aging

**DOI:** 10.3389/fragi.2021.676573

**Published:** 2021-04-12

**Authors:** Consuelo Borrás

**Affiliations:** Freshage Research Group, Department of Physiology, Faculty of Medicine, University of Valencia, Center for Biomedical Research on Frailty and Healthy Aging, INCLIVA Health Research Institute, Valencia, Spain

**Keywords:** lifespan, healthspan, biomarker of aging, animal model of aging, theory of aging

## Lifespan vs. Healthspan

We are currently facing particular challenges related to demographic change. People are reaching very long lives, and the average lifespan has increased considerably since the mid of the twentieth century. The predictions are that citizens older than 65 will increase from 18% of the current population to 28% in 2060. Moreover, citizens with more than 80 years old will increase from 5 to 12% during the same period, becoming as numerous as the young population in 2016 (Christensen et al., 2009).

However, the increase in lifespan has led to a decrease in healthspan, i.e., the period of life free from serious chronic diseases and disabilities (Christensen et al., 2009; Crimmins, 2015). This suggests a situation characterized by an increase in age-related disability and dependency, which will have an impact not only on the well-being and quality of life of the affected people but also on the sustainability of health systems (Murray and Lopez, 2013).

This scenario constitutes the real challenge: unlocking the biological secrets of aging to understand better this process, that will allow to develop adequate interventions to increase not only life but also healthspan and diminish the medical, economic, and social issues associated with old people.

## The Process of Aging

Aging is a very complex process, and therefore there are many definitions to describe it depending on the field involved. From a biological point of view, “aging is a progressive sequence of age-related, widespread, more-or-less common changes observed in every individual of a given species” (Harman, 1988). It is characterized by four postulates (Strehler, 1985; Vina et al., 2007). It is universal: it must occur in all individuals of a species; intrinsic: endogenous factors cause it, although exogenous factors can modulate it; progressive: changes must occur progressively during the lifespan, from early adulthood to the old ages; and deleterious: it arranges negative consequences for the individual.

Aging is not a disease: it is a physiological process that differs from disease because the disease is selective (not universal), intrinsic and extrinsic (not only intrinsic), discontinuous (not progressive), and reversible.

Aging starts early in life, after the development of the organism. It implies that, during many years, many exogenous factors can influence it (accelerators or decelerators of the rate of aging). It may be different in the different individuals, leading to the heterogeneous distinctive of aging. Not all individuals age at the same rate, nor do all organs of the same individual. The complexity of the aging process is the reason for the grand challenge of unlocking its biological secrets.

## Theories of Aging

As aging is multifaceted, many theories are trying to explain the fundamental biological processes underneath it. In 1990 Medvedev claimed that there are more than 300 theories of aging, and the number continues to increase (Medvedev, 1990). This is the natural consequence of the very rapid progress in our understanding of biological phenomena and the application to gerontological research of many new approaches and methods. Almost every major discovery in cell and molecular biology has spawned a new family of aging theories or new advanced versions of older theories (Vina et al., 2013). This same author also commented that the task of reviewing theories of aging has become much more difficult and that a large number of these theories are very selective or outdated. On the other hand, Vijg affirms that some old hypotheses from the beginnings of gerontological science made possible the great scientific revolution in the understanding of aging that is now witnessing (Vijg, 2000). The author agrees with these views and Medvedev's conclusion that the expectation that a truly unified or single-cause theory of aging will emerge is unrealistic. And it is generally accepted that all the pieces of the aging puzzle are not yet available (Troen, 2003). However, we believe that it is possible to offer preliminary solutions to this problem by integrating several complementary theories, classical and modern, which offer logical explanations of the changes occurring in the fundamental levels of biological organization (Vina et al., 2007). In fact, many authors have proposed a unified theory of aging (Kelly, 2011; Barja, 2019).

Thus, we can affirm that there are many theories to explain the aging phenomenon, and even today is not known for sure what the main causes underlying aging are.

## Searching for Good Models of Aging

Aging research can be conducted in many *in vivo* models, which have their own benefits and limitations. Hence, the use of yeasts (*Saccharomyces cerevisiae*), nematodes (*Caenorhabditis elegans*), fruit flies (*Drosophila melanogaster*), short-living fishes (*Nothobranchius furzeri*), rodents (mice and rats), dogs, and non-human primates is common in aging studies and the selected model depends on the objective of the study. Table 1 shows the model's strengths and limitations in aging research (adapted from Folch et al., 2018).

**Table 1:** Animal model's strengths and limitations in aging research.

**Models of Aging**	**Strengths**	**Limitations**
*Caenorhabditis elegans*	Short lifespan. Fast evaluation of interventions. Low costs.	Invertebrate model. Low translationality to humans.
*Drosophila melanogaster*	Short lifespan. Fast evaluation of interventions. Low costs.	Invertebrate model. Low translationality to humans.
*Saccharomyces cerevisiae*	Short lifespan. Fast evaluation of interventions. Low costs.	Invertebrate model. Low translationality to humans.
*Nothobranchius furzeri*	Appropriate for evaluation of interventions	Organs are quite different from those in humans.
Senescence prone inbred strains	Appropriate for evaluation of interventions	Significant differences at a pharmacokinetic level. Lifespan extension could vary between rodent's genders.
Genetically heterogeneous (HET) mouse model	Developed by the National Institute on Aging interventions testing program as the most adequate mammal mice model in aging	Significant differences at a pharmacokinetic level. Lifespan extension could vary between rodent's genders.
Rodent models of progeria	Reduction in time, labor and costs for lifespan studies, as well as the ability to target accelerated aging to specific organs.	Effects of premature aging, not aging itself. Significant differences at a pharmacokinetic level.
Non-human primate models of aging	Best extrapolation of the results to our species.	Expensive. Long time to obtain results.

Models shown in Table 1 are those not genetically modified, but of course, many other models are based on genetic modifications of a specific protein or a protein set that are developed to investigate their role. For example, the Arf/p53 mice model which lives longer demonstrated that p53 is involved in longevity (Matheu et al., 2007). Moreover, there are also models developed for studying aging-related disorders such as cardiovascular, bone, or neurodegenerative disease (Santulli et al., 2015). Finally, some models have been developed to simulate frailty, a clinical syndrome common in the elderly (Howlett and Rockwood, 2014; Santulli et al., 2015), which is based on the decline of their functional capacities with age. One of the best examples of frailty mouse models is the interleukin-10 knockout mouse model since it develops an age-related decline in skeletal muscle strength and similar inflammation and weakness pattern to frailty compared to control mice (Walston et al., 2008).

Animal models have been, are, and will be essential for studying the biological insights of the aging process and developing appropriate interventions. However, successful translation to humans is intricate. It constitutes a challenge and requires several careful considerations, including a proper choice of the animal model, systematic experimental designs, and information integration from bench to bedside.

## Biomarkers of Aging

A biomarker of aging is a biological parameter of an organism that either alone or in some multivariate composite will, in the absence of disease, better predict biological age and functional capacity at some late age than will chronological age (Baker, 1988). The requirements that a biomarker of aging should include are to change progressively with age, to refer to parameters relevant to health and longevity, to be minimally invasive, to be relatively easy to determine, and to be reproducible.

Most of the biomarkers are related to processes and pathways associated with the different theories of aging. As pointed out some years ago by Lopez-Otin et al. (2013), parameters related to the nine hallmarks of aging should be good candidates to be biomarkers of aging, if they meet the requirements mentioned before. Those hallmarks are genomic instability, telomere attrition, epigenetic alterations, loss of proteostasis (and autophagy), deregulated nutrient sensing, mitochondrial dysfunction, cellular senescence, stem cell exhaustion, and altered intercellular communication. Moreover, oxidative stress-related parameters have been also proposed as good candidates as they meet all the requirements for being aging biomarkers (Borras et al., 2003; Ingles et al., 2014). Other good candidates are those parameters related to the so-called process of inflammaging and the immune system function. It is known to decline with age, and many scientists have proposed them as possible aging biomarkers (Martinez De Toda et al., 2016; Fougere et al., 2017; Franceschi et al., 2018).

Although many processes underlying aging are known, and there are many proposed biomarkers of aging related to these processes, there are no fully reliable aging biomarkers. Probably the best approximation to a trustful aging biomarker is that based on a set of several markers. For example, the “epigenetic clock” based on a DNA methylation dataset has enabled accurate age estimates for any tissue across the entire life course (Horvath and Raj, 2018). Indeed, reprogramming the epigenetic clock resets the aging clock, and the organism rejuvenates (Rando and Chang, 2012).

Certainly, a challenge is developing trustful aging biomarkers because it allows a better knowledge of the aging process, and at the same time, it enables developing appropriate interventions to delay aging and promote successful aging.

## Concluding Remarks

We are currently facing particular challenges related to an increased lifespan. However, the increase in lifespan has led to a decrease in healthspan. This scenario constitutes the real challenge: unlocking the biological secrets of aging to understand better this process, that will allow developing adequate interventions to increase not only life but also healthspan and diminish the medical, economic, and social issues associated with old people.

“The molecular mechanisms of aging” specialty section is delved into the basic mechanisms involved in aging to help better understand the aging process. Molecular mechanisms of aging play an integral and interdisciplinary role in modern science and include significant advances in areas including, but not limited to, biomarkers of aging, senescence, altered proteostasis, autophagy, chromosomal alterations, redox system dysregulation, nutrient sensing modulations, genetic and epigenetic changes, mitochondrial energy collapse, intercellular communication alterations, stem cell function dysregulation, and extracellular vesicles alterations.

## Author Contributions

CB wrote the sections of the manuscript and contributed to manuscript revision, read, and approved the submitted version.

## Conflict of Interest

The author declares that the research was conducted in the absence of any commercial or financial relationships that could be construed as a potential conflict of interest.
